# Aggregation propensity of neuronal receptors: potential implications in neurodegenerative disorders

**DOI:** 10.4155/fso.15.39

**Published:** 2015-09-01

**Authors:** Susanna Navarro, Marta Diaz-Caballero, Ricard Illa, Salvador Ventura

**Affiliations:** 1 Institut de Biotecnologia i de Biomedicina, Universitat Autònoma de Barcelona, E-08193 Bellaterra, Spain; 2 Departament de Bioquímica i Biologia Molecular, Facultat de Biociències, Universitat Autònoma de Barcelona, E-08193 Bellaterra, Spain

**Keywords:** aggregation-prone regions, Alzheimer's disease, amyloid, neurodegenerative disorders, neuronal receptor

## Abstract

Misfolding and aggregation of proteins in tissues is linked to the onset of a diverse set of human neurodegenerative disorders, including Alzheimer's and Parkinson's diseases. In these pathologies proteins usually aggregate into highly ordered and β-sheet enriched amyloid fibrils. However, the formation of these toxic structures is not restricted to a reduced set of polypeptides but rather an intrinsic property of proteins. This suggests that the number of proteins involved in conformational disorders might be much larger than previously thought. The propensity of a protein to form amyloid assemblies is imprinted in its sequence and can be read using computational approaches. Here, we exploit four of these algorithms to analyze the presence of aggregation-prone regions in the sequence and structure of the extracellular domains of several neuroreceptors, with the idea of identifying patches that can interact anomalously with other aggregation-prone molecules such as the amyloid-β peptide or promote their self-assembly. The number of amyloidogenic regions in these domains is rather low but they are significantly exposed to solvent and therefore are suitable for interactions. We find a significant overlap between aggregation-prone regions and receptors interfaces and/or ligand-binding sites, which illustrates an unavoidable competition between the formation of functional native interactions and that of dangerous amyloid-like contacts leading to disease.

**Figure 1.**
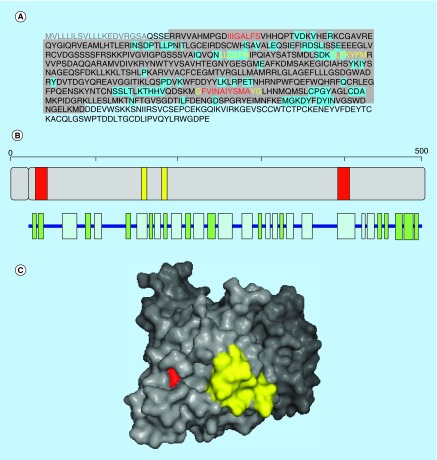
**Aggregation propensity of human GRM5.** **(A)** Receptor's sequence: signal peptide is shown in gray. Regions in the crystal structure are highlighted in gray and those at the interface in blue. **(B)** Length, architecture and secondary structure of the receptor with β-sheets and α-helices shown in green and blue, respectively. **(C)** Surface representation of the crystal structure of the receptor. In **(A–C)** aggregation-prone sequences detected with the consensus of three and four predictors are shown in yellow and red, respectively.

**Figure 2.**
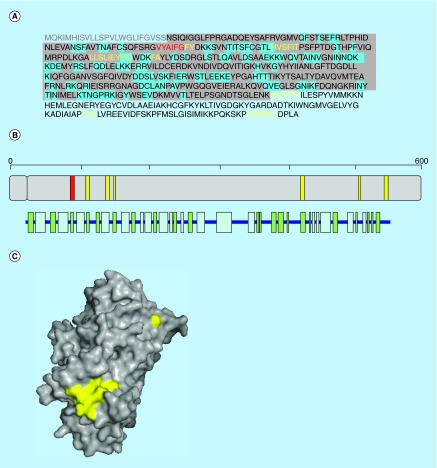
**Aggregation propensity of human glutamate receptor 2.** **(A)** Receptor's sequence: signal peptide is shown in gray. Regions in the crystal structure are highlighted in gray and those at the interface in blue. **(B)** Length, architecture and secondary structure of the receptor with β-sheets and α-helices shown in green and blue, respectively. **(C)** Surface representation of the crystal structure of the receptor. In (A), (B) and (C) aggregation-prone sequences detected with the consensus of three and four predictors are shown in yellow and red, respectively.

**Figure 3.**
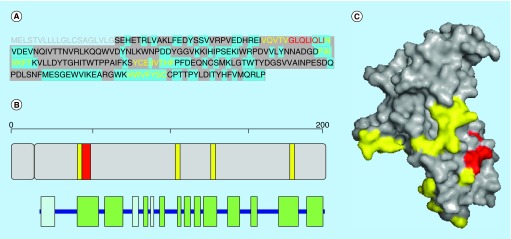
**Aggregation propensity of the acetylcholine receptor α chain.** **(A)** Receptor's sequence: signal peptide is shown in gray. Regions in the crystal structure are highlighted in gray and those exposed to solvent in blue. **(B)** Length, architecture and secondary structure of the receptor with β-sheets and α-helices shown in green and blue, respectively. **(C)** Surface representation of the crystal structure of the receptor. In **(A–C)** aggregation-prone sequences detected with the consensus of three and four predictors are shown in yellow and red, respectively.

**Figure 4.**
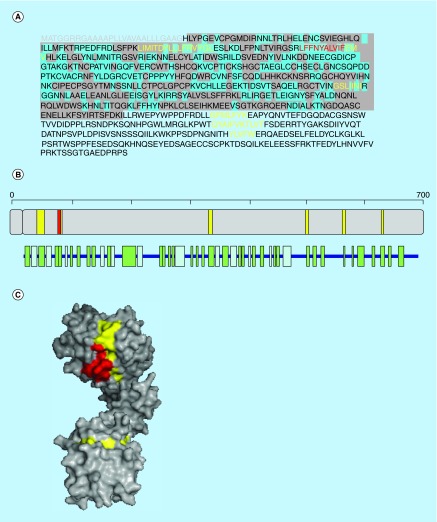
**Aggregation propensity of the human insulin receptor subunit α.** **(A)** Receptor's sequence: signal peptide is shown in gray. Regions in the crystal structure are highlighted in gray and those exposed to solvent in blue. **(B)** Length, architecture and secondary structure of the receptor with β-sheets and α-helices shown in green and blue, respectively. **(C)** Surface representation of the crystal structure of the receptor. In **(A–C)** aggregation-prone sequences detected with the consensus of three and four predictors are shown in yellow and red, respectively.

**Figure 5.**
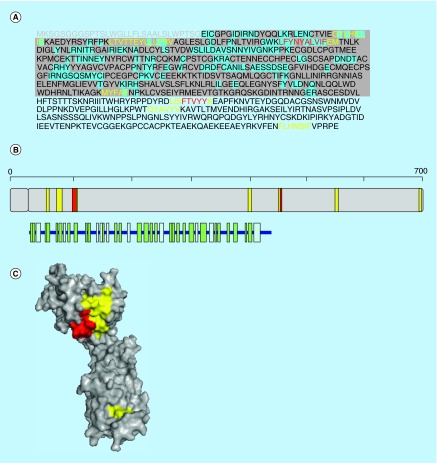
**Aggregation propensity of insulin-like growth factor 1 receptor α subunit.** **(A)** Receptor's sequence: signal peptide is shown in gray. Regions in the crystal structure are highlighted in gray and those at the interface in blue. **(B)** Length, architecture and secondary structure of the receptor with β-sheets and α-helices shown in green and blue, respectively. **(C)** Surface representation of the crystal structure of the receptor. In **(A–C)** aggregation-prone sequences detected with the consensus of three and four predictors are shown in yellow and red, respectively.

## Protein aggregation

Protein misfolding and aggregation is associated with an increasing number of highly debilitating human diseases, including Alzheimer's disease (AD), diabetes and some types of cancer [1]. The proteins involved in these disorders are different and not related in evolutive, sequential or structural terms [2]. However, in all the cases, misfolded conformers of these polypeptides establish non-native intermolecular contacts that result in their deposition into insoluble amyloid aggregates in the intra- or extra-cellular space [3]. Amyloid fibrils are highly ordered and repetitive structures where all polypeptides adopt a common fold [4,5]. They are thread-like protein aggregates with a core region formed by repetitive arrays of β-sheets oriented perpendicularly to the fibril axis forming a structure known as cross-β because it displays two sets of characteristic X-ray diffraction signals, forming a ‘cross’ pattern [6]. These particular fibrils have diameters of approximately 10 nm and often consist of multiple proto-filaments twisted around the fibril axis. Importantly, the ability to form amyloid-like structures is not restricted to a subset of disease-linked proteins and this conformation may be accessed by most, if not all, human proteins, irrespective of their native fold [7]. In fact, the molecular interactions leading to the formation of amyloids are similar to those promoting the folding and functional assembly of proteins. As a result, folding and aggregation pathways are continuously competing in the cell [8,9]. This implies that the proteins involved in conformational disorders might me much larger than previously thought [10], neuronal receptors among them.

## Prediction of protein aggregation

Although any polypeptide has the potential to self-assemble into β-sheet-enriched amyloid-like conformations [7], the composition and the primary structure of a protein strongly influences its propensity to aggregate [11]. Not all the regions in a protein contribute equally to its aggregation behavior and there exist short specific amino acid stretches with defined physicochemical properties able to initiate the self-assembly process by nucleating the aggregation reaction [12]. Small sequential changes inside or close to these sequences usually have a large impact on protein solubility [13,14]. Moreover, short peptides corresponding to these regions are able to form amyloids, in the absence of the rest of the protein sequence [15,16]. The main intrinsic features that modulate the protein-aggregation propensity of proteins have been identified. They have been exploited by different prediction algorithms to detect β-aggregating motifs in protein sequences and to infer their experimental aggregation rates [17]. We will use several of these algorithms to forecast the aggregation propensities of different neuronal receptors.

## Aβ peptide aggregation in AD

AD is a complex neurodegenerative multifactorial pathology characterized by a progressive and irreversible decline of cognitive function. It is the most common form of dementia in individuals over 65 years of age; and the incidence of AD doubles every 5 years beyond the age of 65. According to the World Alzheimer Report 2011, it is estimated that 36 million people worldwide suffer from dementia, and patients are expected to double every 20 years to 66 million by 2030, and 115 million by 2050 [18]. A majority of AD is sporadic, although several genetic linkages have also been identified.

A hallmark of AD is the extracellular accumulation of aggregated amyloid-β (Aβ). Aβ is a 37–43 residues peptide that results from multiple proteolytic cleavage of a large transmembrane precursor, the amyloid precursor protein [19]. AD pathological features result from a chronic imbalance between Aβ production and clearance. The self-assembly of initially soluble but sticky Aβ molecules into oligomers and large amyloid fibrils initiate the pathogenic cascade. The process of Aβ amyloid fibril formation is a multistep process that follows a nucleation-elongation mechanism in which the formation of the first oligomeric assemblies is the rate limiting step of the reaction and is followed by a rapid fibril elongation phase to form protofibrils and fibrils [20]. Despite the toxic effect was initially associated to the presence of mature fibrils, it is now clear that the smaller intermediate species are also toxic for neurons. In fact it is becoming evident that AD is initiated by alterations in hippocampal synaptic function caused by diffusible oligomeric assemblies of Aβ. These early extracellular aggregates exhibit hydrophobic aggregation-prone regions to solvent that can interact anomalously with different cellular components, and specifically with cellular membranes promoting oxidative stress and Ca^2+^ homeostasis deregulation [21]. It has been proposed recently that apart from interacting with the lipidic membrane components, aggregated Aβ can interact with proteins anchored in such membranes, including neuronal receptors [22]. Such interactions might result in signaling dysfunction or even in the sequestration of the receptor into the amyloid aggregate with deleterious effects for neurons. In fact, since Aβ and receptors share the endocytic pathways it is also possible that wrong interactions would occur during traffic.

## Prediction of aggregation prone regions in neuronal receptors

To address if human neuronal receptors display aggregation-prone regions susceptible of interacting with Aβ oligomeric assemblies, we have selected five different receptors for which the 3D structure were available. We have used human structures in most cases, when these were not available we used homolog proteins with a high sequential identity. In all cases we have analyzed the extracellular domains of the receptors, which include the ligand-binding sites, because it is more likely that Aβ diffusible oligomeric species will interact with them than with domains already embedded in the membrane. We have used PDBePISA (Protein Interfaces, Surfaces and Assemblies) [23] to deconstruct oligomeric receptors into their monomers in such a way that we can identify which residues are located at the interface and therefore might become exposed to solvent when the domain dissociates.

To have a complete description of the aggregation properties of the selected neuronal receptors we have used four different predictors based on different concepts and calculation schemes:

Waltz [24] uses a position-specific scoring matrix deduced from the biophysical and structural analysis of the amyloid properties of a large set of hexapeptides to distinguish between amyloid and amorphous aggregating sequences [25].
Tango [26] is based on the analysis of the physico-chemical principles underlying β-sheet formation together with the assumption that the core regions of an aggregated polypeptide are protected from the solvent [27].
Aggrescan [28] is based on an aggregation propensity scale for natural amino acids derived from *in vivo* experiments in bacteria and on the assumption that short and specific sequence stretches modulate protein aggregation [29,30].
Amylpred [31] is a consensus predictor of amyloid propensity that integrates the results of five different methods in a single output to detect the regions in a protein with the highest intrinsic propensity to form amyloids [32].

As a stringent criterion for identification of aggregation-prone regions we have considered as positive hits only those regions recognized simultaneously by at least three of these algorithms.

## Aggregation-prone regions in GRM5

Human GRM5 (P41594) is a G-protein coupled receptor for glutamate. The binding of the ligand to the receptor promotes a conformational change that initiates the signaling pathway through guanine nucleotide-binding proteins, which further modulate down-stream effectors. Signaling involves a phosphatidylinositol-calcium second messenger system, which triggers a calcium-activated chloride current. GMR5 has been shown to be a co-receptor for Alzheimer Aβ oligomers bound to cellular prion protein [33].

GRM5 is a receptor of 1212 residues located at the cell membrane, residues 22 to 579 correspond to the extracellular region, exhibiting an α β 3-layer (aba) sandwich structure, with 32% of helical structures (16 helices; 159 residues) and 18% of β-sheets (22 strands; 91 residues) (PDB 3LMK).

Four aggregation regions comprising residues 35–45 (IIIGALFSV), 161–166 (LLQLFN), 183–198 (TLFKYFM) and 398–410 (GFVINAIYSMAYG) are detected with the consensus of three predictors (Figure 1). The second and fourth sequence stretches are located at two helices (α-helices 3 and 14), the first one in β-strand 2 and the third one in a turn. The second and third aggregation-prone regions overlap with the interface between domains in the receptor, with 83 and 42% of their residues being involved in interdomain contacts. The first and fourth stretches are totally hidden in the inner core in the native structure of the receptor. The four predictors coincide that the sequences 35-IIIGALFS-42 and 399-FVINAIYSMA-408 are amyloidogenic.

## Aggregation-prone regions in human glutamate receptor 2

Human glutamate receptor 2 (AMPA 2) (P42262) plays a central role in excitatory synaptic transmission at the CNS. AMPA 2 is a receptor for glutamate that acts as ligand-gated ion channel. Binding of the excitatory neurotransmitter l-glutamate to the receptor promotes a structural change that forces the opening of the cation channel. This action transforms the chemical input into an electrical signal, upon which the receptor desensitizes and adopts a transient inactive conformation, in which it remains bound to an agonist. This receptor is activated by the presence of Aβ oligomers at the synapsis [34].

AMPA 2 is a 883 residues receptor which sequence is further processed into a mature form (25–883). The crystallized extracellular region corresponds to residues 25 to 395, showing an α β 3-layer (aba) sandwich structure, with 34% helical structure (13 helices; 135 residues) and 21% of β sheet (18 strands; 82 residues) (PDB 2WJW) [35].

Three hot spots of aggregation comprising residues 85–92 (VYAIFGFY), 108–113 (HVSFIT) and 136–144 (LLSLIEYYQ) are detected in the crystallized receptor (Figure 2). The first and the second sequence stretches are located at two parallel strands (β-strand 3 and 4), the third one at the α-helix 4. This last region is exposed and overlaps significantly with the interface between extracellular domains in their dimer structure (Figure 2). The sequence 85-VYAIG-90 is an amyloidogenic region according to the four predictors, but is essentially buried inside the native structure. Other three aggregation-prone regions comprising residues 415–420 (TVVVTT), 500–503 (LTIT), 534–539 (GVFSFL) are predicted outside the crystallized domain. In agreement with data on GRM5, the glutamate binding sites do not correspond to any of the above-described aggregation-prone regions.

## Aggregation-prone regions in acetylcholine receptor

After binding to acetylcholine the mouse acetylcholine receptor (AChR) responds with an extensive change in conformation that affects all their subunits and leads to opening of an ion-conducting channel across the cellular membrane. It exhibits a pentameric structure with two α chains (P04756), and three other structurally different subunits. This receptor interacts directly with Aβ oligomers [36].

The α chain of AChR is 457 residues long. After signal peptide processing the AChR sequence mature form comprises residues 21–457, with residues 21–230 located at the extracellular domain. Extracellular mature α AChR shows 8% helical structure (three helices; 18 residues) and 47% of β sheets (14 strands; 100 residues) and is a mainly β distorted sandwich (PDB: 2QC1). Mouse α AChR shares 83.6% identity with the human AChR α receptor chain [37].

Four regions are predicted as aggregation prone with the consensus of three predictors, comprising residues 49–62 (VQVTVGLQLIQLIN), 147–155 (YCEIIVTHF) (Figure 3). In this mainly β protein the first, second and fourth sequences are located at β-sheets (β-sheets 2, 8 and 12) and the third corresponds to a β turn. The solvent exposed degree of the first and second aggregation-prone regions is 28.57 and 44.44%, and interestingly, regions 120–126 (FAIVKFT) and region 206–212 (HWVFYSC) are fully exposed to solvent. It is likely that in native conditions these dangerous regions are covered by interactions with other domains of the receptor. The four predictors coincide that 54-GLQLIQLI-61 is amyloidogenic and exposed to solvent.

## Aggregation-prone regions in human insulin receptor

Human insulin receptor subunit α (P06213) is one of the subunits of the insulin receptor (IR). This transmembrane receptor is activated by insulin and belongs to the tyrosine receptors superfamily. Binding of insulin results in the phosphorylation of several intracellular substrates, which act as docking proteins for other signalling polypeptides. From a metabolic point of view, the IR is responsible for the regulation of glucose homeostasis and its involved in a wide range of pathologies, including diabetes and cancer. Aβ oligomers have been shown to reduce responsiveness to insulin in synapsis [38].

Human IR is a 1382 amino acid residues receptor which sequence is further processed into IR subunit α (28–758). The crystallized subunit α comprises residues 25–457, which show 11% helical structure (9 helices; 56 residues) and 29% of β-sheets (43 strands; 141 residues) (PDB 2HR7). Two different structural domains are evident in the crystallized chain: an α β horseshoe (4–191, 310–468) and a mainly β ribbon domain (192–309).

Several hot spots of aggregation are detected with the consensus of three predictors, 54–69 (LIMITDYLLLFRVYGL), 87–99 (LFFNYALVIFEMV), 338–344 (GSLIINI), 502–508 (GFMLFYK), 561–571 (QYAIFVKTLVT), 628–632 (GVFSFL) (Figure 4). The three first sequence stretches correspond to parallel β-strand structures, with 43.75, 69.23 and 28.57% of their residues exposed to solvent, respectively. The other three aggregation-prone regions are located in structurally undefined protein segments. Interestingly enough, in this receptor the binding site for insulin overlaps with the first and second aggregation sequence stretches. The sequence 87-LFFNYALVIF-96 in the second stretch is predicted as highly amyloidogenic.

## Aggregation-prone regions in IGF1R

Human IGF1R (P08069) is, as IR, a member of the tyrosine kinase receptors. The activated form of this receptor is involved in cell growth and survival control. In addition, it is an important determinant of tumor transformation and the survival of malignant cells in different cancers. Binding of the ligand results in activation of the receptor kinase and receptor autophosphorylation, as well as the phosphorylation of tyrosine residues in different protein substrates, that function as signaling adapter proteins. Aβ oligomers have been shown to mediate IGF-1R activation in the hippocampus of AD brain during the early stages of disease development [39].

IGR1R complete sequence has 1367 residues, which further processing renders the α subunit located at the extracellular environment comprising residues 31–736. The crystallized IGR1R α subunit exhibits three structural domains, (1–183) α/β horseshoe, (224–299), mainly β ribbon domain and, (300–478) another α/β horseshoe domain (PDB 1IGR).

Seven aggregation prone regions are detected with the consensus of three predictors, comprising residues 27–35 (GYLHILLIS), 48–60 (LTVITEYLLLFRV), 81–92 (LFYNYALVIFEM), 416–420 (MYFAF), 490–498 (LISFTVYY), 546–551 (QYAVYV) and 695–701 (FLHNSIF) (Figure 5). The crystal structure includes residues 1–478, being the first, second, third and fourth sequence stretches located at β-strands 3, 5 and 8 in the first β-sheet and at β-strand 8 in the ninth β-sheet, respectively. The main property that differentiates IGF1R and other members of the IR family from other receptor families is their presence at the cell surface as disulfide-linked dimers and require domain conformational changes rather than oligomerization for signaling. The first four aggregation-prone regions show a 55.55, 23.08, 66.67 and 20% of their residues exposed to solvent and might be therefore involved in inter-domain contacts in the dimeric structure or in functional binding. In fact, residues Y28, H30 and L33 in the first region and L696, H697, N698, Y100 and Y101 in the last aggregation stretch have been shown to be crucial for ligand binding. Sequences 81-LFYNYALVIF-90 and, 493-FTVYY-497 are predicted as amyloidogenic with the consensus of the four predictors.

## Conservation of aggregation prone-regions in neuronal receptors among different species

To decipher whether the detected aggregation-prone sequences stretches might play a conserved role in different organisms we made multiple alignments of the sequences of the five analyzed receptors in vertebrates. As an example we show the results in Table 1 for the α chain of the acetylcholine receptor. As a general rule, independently, of the structural location of the aggregating stretches, they are highly conserved among species, and most of the substitutions have a conservative character, with isosteric and isopolar changes in most cases. Provided that we know now that there is a strong selective pressure against the maintenance of aggregation-prone regions in protein sequences along evolution, especially when they become exposed to solvent in the folded structure, the strict conservation of the detected stretches along vertebrate sequences clearly indicate that they play an important structural and/or functional role in the respective receptors.

## Conclusion & future perspective

Despite the work presented here is based on *in silico* predictions, which should be further validated experimentally, our data suggest that, as previously demonstrated for other globular proteins, the extracellular domains of neuronal receptors cannot skip the presence of aggregation-prone regions in their sequences. However, the number and size of these stretches is rather small if we compare them with the average in globular proteins. This is likely a consequence of their important physiological role, since it has been shown that essential proteins are under higher selective pressure against the accumulation of aggregating sequences that nonessential ones. The detected regions are highly conserved, pointing out to an important role in the function or the structure of these receptors. Importantly, and contrary to what one could expect, many of these regions appear to be exposed to solvent in the monomeric forms. A structural inspection indicates that they are part of the interface between subunits or even overlap with the binding site for proteinic substrates. These data confirm that protein–protein interaction surfaces and regions with high aggregation propensity overlap significantly in proteins. The result is that formation of native receptor oligomers or native complexes with substrates and self-aggregation reactions or anomalous interactions with other aggregating ensembles such as Aβ oligomers are probably constantly competing in the extracellular face of neuronal cells, which might result in signaling problems. Moreover, neurons are vulnerable to perturbations in the endocytosis process in which they recycle their receptors. It is likely that Aβ oligomers might interact promiscuously with the aggregation-prone regions in conformational flexible, monomeric and unliganded receptors during this process, impeding them to arrive to their proper destinations in the neuronal membrane.

**Table 1.** **Sequence alignment of detected aggregation-prone regions in the α chain of the acetylcholine receptor in different species.**

***Mus musculus***	**VQVTVGLQLIQLIN**	**FAIVKFT**	**YCEIIVTHF**	**HWVFYSC**
*Bos Taurus*	V**E**VTVGLQLIQLIN	FAIVKFT	YCEIIVTHF	HWVFY**A**C
*Canis lupus familiaris*	VQVTVGLQLIQLIN	FAIVKFT	YCEIIVTHF	HWVFY**A**C
*Cricetulus griseus*	VQVTVGLQLIQLIN	FAIVKFT	YCEIIVTHF	HWVFYSC
*Heterocephalus glaber*	VQVTVGLQLIQLIN	FAIVKFT	YCEIIVTHF	HWVFYSC
*Homo sapiens*	VQVTVGLQLIQLIN	FAIVKFT	YCEIIVTHF	H**S**V**T**YSC
*Pan troglodytes*	VQVTVGLQLIQLIN	FAIVKFT	YCEIIVTHF	H**S**V**T**YSC
*Sus scrofa*	VQVTVGLQLIQLIN	FAIVKFT	YCEIIVTHF	H**R**V**L**Y**A**C
*Torpedo marmorata*	V**DI**TVGLQLIQLIN	FAIV**HM**T	YCEIIVTHF	HWVYY**T**C

Residues that diverge from the mouse sequence, for which the crystal structure has been solved, are bold.

**Executive summary**

The formation of amyloid-like structure is an intrinsic property of most proteins and polypeptides.
The presence of aggregation-prone regions can be read out directly from the primary protein sequence.
Neuronal receptors exhibit aggregation-prone regions exposed to the solvent that might interact with hydrophobic diffusible amyloid-β peptide oligomers.
Aggregation-prone regions in neuronal receptors often overlap with the interface between monomers or domains or coincide with the ligand-binding sites, illustrating a competition between functional and anomalous interactions.
